# Supplementary material for: MetaRibo-Seq measures translation in microbiomes
Source: Nat Commun. 2020 Jun 29;11:3268. doi: 10.1038/s41467-020-17081-z (PMC7324362; doi:10.1038/s41467-020-17081-z)
Supplement: Supplementary file 10 — Supplementary Data 7 [file 41467_2020_17081_MOESM10_ESM.zip › File2/Confidence_VeryHigh_Taxonomy/394515_out.krona.html]

Javascript must be enabled to view this page.

members
magnitude
magnitudeUnassigned
count
unassigned
taxon
rank

394515\_out

6

2
superkingdom
6

phylum
1239
6

6
class
186801

186802
order
6

541000
family
6

genus
1263
6

5
species

SRS893230\_contig\_number\_1425SRS893230\_contig\_number\_1496SRS893230\_contig\_number\_1496SRS893230\_contig\_number\_8177SRS893230\_contig\_number\_contig-100\_5852.60442
1262963

1

SRS024331\_contig\_number\_contig-100\_5.208580
2293243
species
